# Perceptions of radiologists on structured reporting for cancer imaging—a survey by the European Society of Oncologic Imaging (ESOI)

**DOI:** 10.1007/s00330-023-10397-6

**Published:** 2024-01-11

**Authors:** Doris Leithner, Evis Sala, Emanuele Neri, Heinz-Peter Schlemmer, Melvin D’Anastasi, Michael Weber, Giacomo Avesani, Iztok Caglic, Damiano Caruso, Michela Gabelloni, Vicky Goh, Vincenza Granata, Wolfgang G. Kunz, Stephanie Nougaret, Luca Russo, Ramona Woitek, Marius E. Mayerhoefer

**Affiliations:** 1 Department of Radiology, NYU Grossman School of Medicine, New York, NY USA; 2 Department of Radiology, Universita Cattolica del Sacro Cuore, Rome, Italy; 3 Advanced Radiology Center, Fondazione Universitario Policlinico A. Gemelli IRCCS, Rome, Italy; 4 Diagnostic and Interventional Radiology, Department of Translational Research, University of Pisa, Pisa, Italy; 5 Department of Radiology, German Cancer Research Center, Heidelberg, Germany; 6 Medical Imaging Department, Mater Dei Hospital, University of Malta, Msida, Malta; 7 Department of Biomedical Imaging and Image-guided Therapy, Medical University of Vienna, Vienna, Austria; 8 Department of Radiology, Radiation Oncology and Hematology, Fondazione Policlinico Universitario, A. Gemelli IRCCS, Rome, Italy; 9 Department of Radiology, Addenbrooke’s Hospital and University of Cambridge, Cambridge, UK; 10 Department of Medical Surgical Sciences and Translational Medicine, Sapienza University of Rome, Sant’Andrea University Hospital, Rome, Italy; 11 Nuclear Medicine Unit, Department of Translational Research, University of Pisa, Pisa, Italy; 12 School of Biomedical Engineering & Imaging Sciences, King’s College London, London, UK; 13 Department of Radiology, Guy’s & St Thomas’ Hospitals NHS Foundation Trust, London, UK; 14 Division of Radiology, Istituto Nazionale Tumori IRCCS Fondazione Pascale-IRCCS, Naples, Italy; 15 Department of Radiology, University Hospital LMU Munich, Munich, Germany; 16 Department of Radiology, IRCM INSERM U1194, SIRIC, Montpellier, France; 17 Research Centre for Medical Image Analysis and Artificial Intelligence, Danube Private University, Krems, Austria

**Keywords:** Medical oncology, Diagnostic imaging, Quality improvement

## Abstract

**Objectives:**

To assess radiologists’ current use of, and opinions on, structured reporting (SR) in oncologic imaging, and to provide recommendations for a structured report template.

**Materials and methods:**

An online survey with 28 questions was sent to European Society of Oncologic Imaging (ESOI) members. The questionnaire had four main parts: (1) participant information, e.g., country, workplace, experience, and current SR use; (2) SR design, e.g., numbers of sections and fields, and template use; (3) clinical impact of SR, e.g., on report quality and length, workload, and communication with clinicians; and (4) preferences for an oncology-focused structured CT report. Data analysis comprised descriptive statistics, chi-square tests, and Spearman correlation coefficients.

**Results:**

A total of 200 radiologists from 51 countries completed the survey: 57.0% currently utilized SR (57%), with a lower proportion within than outside of Europe (51.0 vs. 72.7%; *p* = 0.006). Among SR users, the majority observed markedly increased report quality (62.3%) and easier comparison to previous exams (53.5%), a slightly lower error rate (50.9%), and fewer calls/emails by clinicians (78.9%) due to SR. The perceived impact of SR on communication with clinicians (i.e., frequency of calls/emails) differed with radiologists’ experience (*p* < 0.001), and experience also showed low but significant correlations with communication with clinicians (*r* = − 0.27, *p* = 0.003), report quality (*r* = 0.19, *p* = 0.043), and error rate (*r* = − 0.22, *p* = 0.016). Template use also affected the perceived impact of SR on report quality (*p* = 0.036).

**Conclusion:**

Radiologists regard SR in oncologic imaging favorably, with perceived positive effects on report quality, error rate, comparison of serial exams, and communication with clinicians.

**Clinical relevance statement:**

Radiologists believe that structured reporting in oncologic imaging improves report quality, decreases the error rate, and enables better communication with clinicians. Implementation of structured reporting in Europe is currently below the international level and needs society endorsement.

**Key Points:**

*• The majority of oncologic imaging specialists (57% overall; 51% in Europe) use structured reporting in clinical practice.*

*• The vast majority of oncologic imaging specialists use templates (92.1%), which are typically cancer-specific (76.2%).*

*• Structured reporting is perceived to markedly improve report quality, communication with clinicians, and comparison to prior scans.*

**Supplementary Information:**

The online version contains supplementary material available at 10.1007/s00330-023-10397-6.

## Introduction

Structured reporting (SR) is regarded as a key element in strengthening the radiologist’s role in medicine, providing both referring physicians and patients with the best quality of service. Its use is increasing worldwide, with support from large international specialty societies such as the European Society of Radiology (ESR) and the Radiological Society of North America (RSNA) [1–5]. SR is essential in radiology and oncologic imaging, as it enables standardized and structured documentation of findings by ensuring diagnostic imaging report completeness, utility, and comparability for longitudinal scans, ideally also between institutions [6–8]. SR also has the potential to facilitate communication and decision-making among radiologists and clinicians alike, especially when combined with standardized quantitative and qualitative terminology (for instance, for number of lesions, diagnostic confidence, and changes in lesion size), aiming to increase healthcare quality and improve the workflow. For instance, SR has been shown to decrease rates of report errors while increasing clarity and comprehensiveness [9–11]. Furthermore, the standardization of report structure in combination with standardized terminology and quantitative metrics may facilitate integration with other diagnostic and clinical parameters, as well as data mining for research and clinical purposes [12, 13].

While SR appears to be regarded favorably among radiologists and clinicians [14–18], its widespread implementation remains challenging due to the current heterogeneity in terms of report structure and contents (i.e., lack of national and international standards), technical aspects (e.g., graphical user interface vs. document-based solutions), and its major impact on radiologists’ daily practice [19]. In addition, the field of radiology faces multiple changes such as increased numbers of examinations (as shown by OECD data [20]), radiologist shortage and burnout [21], a shift towards teleradiology services, as well as the application of artificial intelligence (AI) [6]. Notably, the integration of AI with SR has particular potential to enhance remote reporting capabilities and workflow efficiency. In particular, natural language processing (NLP), a branch of AI applying computer-based techniques to analyze speech or text, has various applications in radiology reporting, such as automatic assignment of clinical scores (e.g., tumor stage, or RADS scores), decision support, and conversion into SR [22].

Since cancer frequently presents as a whole-body, multi-organ disease, the field of oncologic imaging could particularly benefit from SR and standardization. For instance, SR has the potential to facilitate comparison between serial reports for radiologists, allow faster access to key findings such as resection margins, and provide a better overview of tumor spread and burden as well as treatment response for clinicians [15, 23–25].

The aim of our survey was therefore to determine and analyze radiologists’ current use of, and experience with, SR in oncologic imaging, and based on it, to provide recommendations for a tailored oncologic structured report. With this, the European Society of Oncologic Imaging (ESOI) strives to contribute, in an international effort of the cancer imaging community, to SR development and its more widespread use in clinical practice.

## Materials and methods

### Survey design and distribution

Our study was based on a survey among oncologic imaging specialists; no patient data were included, and therefore, Institutional Review Board approval was not required. Oncologic imaging was broadly defined as diagnostic imaging in patients with known or suspected cancers for the purpose of baseline or follow-up (i.e., post-treatment) disease extent evaluation, using radiology- and nuclear medicine-based tests. Two ESOI Research Committee members (D.L. and M.E.M.) drafted survey questions. The questionnaire was validated through an open review by ten members of the Research Committee of ESOI, using Google Docs. Since this committee represents a peer group for the investigated topic, the questions included were considered to provide content validity. A pilot test was performed by five external radiologists. Following review and amendments, the final survey was created on Google Forms and sent via email by the ESOI office to ESOI active full members for 2022 (*n* = 799). The questionnaire was available from July 7, 2022, to August 23, 2022, for a total of 48 days. Two reminders were sent to encourage participation.

The survey comprised 28 questions in total, with a combination of multiple and single choices and free text, and was organized into four main sections (Table 1). The first section gathered general participant information, such as country, years of radiology practice, and work setting, as well as a question inquiring about the current use of SR. The second and third sections addressed exclusively those survey participants currently using SR. The second and third sections addressed exclusively those survey participants who were currently using SR. The second section aimed to gather in-depth information on the design of SR, and included, for example, the number of sections and fields (i.e., anatomic regions), the use and design of templates, standardized language, and tailoring to specific cancer types. In the third section, we questioned participants about the perceived implications of SR for reporting (e.g., perceived quality and reading time), workflow, and communication with clinicians. The final section, which again addressed all survey participants, asked for radiologists’ preferences for an oncology-focused structured report for CT chest/abdomen/pelvis, given that this is currently the most widely performed whole-body cross-sectional imaging test in clinical practice. The full survey questions are reported in Table 1.

**Table 1:** Survey questions

I. General Information
1. Which country do you work in?
2. Are you board-certified or fellowship trained? a. Yes b. No
3. In which setting do you work? a. Public Hospital, non-academic b. Private Hospital, non-academic c. University hospital/academic center d. Private practice
4. How many years have you practiced radiology (including residency)? a. < 6 years b. 6 –10 years c. 10–20 years d. > 20 years
5. Do you use structured reporting in your daily practice? a. Yes b. No, but I would like to or plan to use it c. No, and I do not want to use it
If your answer to question 5 was Yes, please proceed to part II. Otherwise, proceed to part IV.
II. Design of Structured Reports
6. How many sections (e.g., Indication, Technique, Findings, Summary) do your structured reports typically have? a. 3 b. 4 c. 5 d. 6 e > 6
7. How many fields (anatomic regions) does the Findings section have for whole-body cross-sectional imaging (e.g., CT chest/abdomen/pelvis)? a. None – free text b. 2–5 c. 6 –10 d. 11–15 e. > 15
8. Do you use a template for your structured report? a. Yes, document-based b. Yes, form-based (e.g., clickable graphical user interface) c. No
9. For which imaging techniques do you use templates (select all that apply)? a. CT b. MRI c. Ultrasound d. PET e. Other
10. Are templates tailored to the type of cancer, and if so, for which imaging techniques (select all that apply)? a. CT b. MRI c. Ultrasound d. PET e. None
11. If you use cancer-specific templates, for which types of cancer (select all that apply)? a. Head/Neck b. Lung c. Breast d. Hepatobiliary e. Pancreas f. Kidney/urinary tract g. GI tract h. Gynecological i. Hematological j. Musculoskeletal k. Other: free text
12. Are the fields of your structured report pre-filled (e.g., with “unremarkable”)? a. Yes, and active confirmation required for each field (checkbox etc.) b. Yes, but active confirmation not required c. No
13. Do structured reports across your institution use standardized language, e.g. for number of lesions (e.g., few, multiple, numerous) or likelihood of malignancy (e.g., possibly, probably, likely)? a. Yes b. No
14. Do you use unidimensional or bidimensional measurements? a. Unidimensional b. Bidimensional c. Both of the above
15. Does your structured report Summary section provide tumor stage (e.g., TNM, FIGO) for baseline scans, or treatment response (e.g., complete remission, progression) for follow-up scans? a. Tumor stage for baseline scans b. Treatment response for follow-up scans c. Tumor stage and treatment response d. Neither tumor stage nor treatment response
III. Impact of structured reporting
16. How did structured reporting affect report quality? a. Increased markedly b. Increased slightly c. Unchanged d. Decreased slightly e. Decreased markedly
17. How did structured reporting affect report length? a. Increased markedly b. Increased slightly c. Unchanged d. Decreased slightly e. Decreased markedly
18. How did structured reporting affect your perceived workload? a. Increased markedly b. Increased slightly c. Unchanged d. Decreased slightly e. Decreased markedly
19. How did structured reporting affect reading times (time to report finalization)? a. Increased markedly b. Increased slightly c. Unchanged d. Decreased slightly e. Decreased markedly
20. How did structured reports affect comparison to previous exams? a. Markedly easier b. Slightly easier c. Unchanged d. Slightly more difficult e. Markedly more difficult
21. How did structured reporting affect radiologists’ error rate? a. Markedly lower b. Slightly lower c. Unchanged d. Slightly higher e. Markedly higher
22. How did structured reporting affect communication with clinicians? a. Markedly fewer calls/emails b. Slightly fewer calls/emails c. Unchanged d. Slightly more calls/emails e. Markedly more calls/emails
IV. Suggestions for ESOI structured report template for CT chest/abdomen/pelvis
23. How many fields (anatomic regions) should the Findings section have? a. None – free text b. 2 –5 c. 6 –10 d. 11–15 e. > 15
24. Should standardized language be used, and a syllabus included? a. Yes, for number of lesions b. Yes, for likelihood of malignancy c. Yes, for number of lesions and likelihood of malignancy d. No
25. Should tumor stage (e.g., TNM, FIGO) be provided for baseline scans? a. Yes b. No
26. Should treatment response category (CR, PR, SD, PD) be provided for follow-up scans? a. Yes b. No
27. Should baseline data always be included for comparison / to determine response? a. Yes b. No
28. Additional comments and suggestions

### Statistical analysis

Following data collection from the survey participants, data cleaning was performed by the first and senior author to check for data entry errors. We primarily used descriptive statistics, including bar graphs, to report and display results. In addition, we used Pearson chi-square tests to identify possible significant differences in SR utilization between European and non-European respondents (according to the definition of the National Geographic Society), and between academic and non-academic radiologists.

In addition, in the subgroup of SR users, we used chi-square tests to determine associations of two survey participant descriptors (work setting and radiologists’ experience) and two SR design features (template use and use of standardized language) with the eight descriptors of SR impact (i.e., on quality, report length, workload, reading times, comparison to prior, error rate, and communication with clinicians). Spearman correlation coefficients (r) were used to assess relationships between radiologists’ experience (as an ordinal, categorical variable) and the above descriptors of perceived SR impact.

We did not perform a dedicated reliability analysis, because each question represents a separate topic (i.e., content is measured only once). Therefore, neither measures for internal consistency, test-retest, nor inter-rater agreement were applicable.

All analyses were performed using SPSS 28.0.1 (IBM). The specified level of significance was *p* < 0.05.

## Results

### Participant characteristics and use of SR

The survey was completed by 200 radiologists from 51 countries (Table 2), predominantly from Europe (72.5%, *n* = 145). The vast majority of respondents were board-certified or fellowship-trained (92.5%, *n* = 185). With 57.5% (*n* = 115), most respondents worked in a university hospital or other academic center, 22.0% (*n* =44) in non-academic public hospitals, 11.0% (*n* = 22) in non-academic private hospitals, and 9.5% (*n* = 19) in private practices. A minority of 7.5% (*n* = 15) respondents had practiced radiology, including residency, for < 6 years, 19.0% (*n* = 38) for 6–10 years, 35.5% (*n* = 71) for 11–20 years, and 38.0% (*n* = 76) for > 20 years.

**Table 2:** Country of origin of survey participants

	*n*	%		*n*	%
Australia	4	2.0	Netherlands	4	2.0
Austria	7	3.5	New Zealand	1	0.5
Belarus	1	0.5	Nigeria	3	1.5
Belgium	3	1.5	Norway	4	2.0
Brazil	1	0.5	Poland	8	4.0
Bulgaria	1	0.5	Portugal	3	1.5
Cameroon	2	1.0	Romania	10	5.0
Canada	1	0.5	Russia	1	0.5
Chile	2	1.0	Serbia	1	0.5
China	1	0.5	Slovakia	3	1.5
Colombia	3	1.5	Slovenia	1	0.5
Croatia	6	3.0	South Africa	12	6.0
Czech republic	1	0.5	Spain	9	4.5
Denmark	3	1.5	Sweden	3	1.5
Egypt	1	0.5	Switzerland	2	1.0
France	1	0.5	Turkey	4	2.0
Germany	9	4.5	Uganda	1	0.5
Greece	11	5.5	Ukraine	3	1.5
Hungary	1	0.5	UK	16	8.0
India	3	1.5	Uruguay	2	1.0
Israel	1	0.5	USA	4	2.0
Italy	27	13.5			
Kazakhstan	2	1.0			
Kenya	3	1.5			
Latvia	1	0.5			
Lithuania	1	0.5			
Malaysia	2	1.0			
Malta	2	1.0			
Mexico	3	1.5			
Morocco	1	0.5			

The majority of radiologists utilized SR in their daily practice (57.0%, *n* = 114), followed by those wishing or planning to use it in the future (38.5%, *n* = 77); only a small minority were current non-SR users with no interest in using SR in the future (4.5%, *n* = 9). A lower percentage of European survey responders (51%, *n* = 74/145) used SR in their daily practice, compared to non-Europeans (72.7%, *n* = 40/55); this difference was statistically significant (*p* = 0.006). Within Europe, the percentage of participating academic radiologists was higher (60.0%, *n* = 87/145) than outside of Europe (50.9%, *n* = 28/55), although not significantly (*p* = 0.25). There was also no significant difference in the percentage of SR users between academic (57.4%, *n* = 66/115) and non-academic (56.5%, *n* = 48/85) settings (*p* = 0.90).

### Design of structured reports and templates

**Fig. 1:**
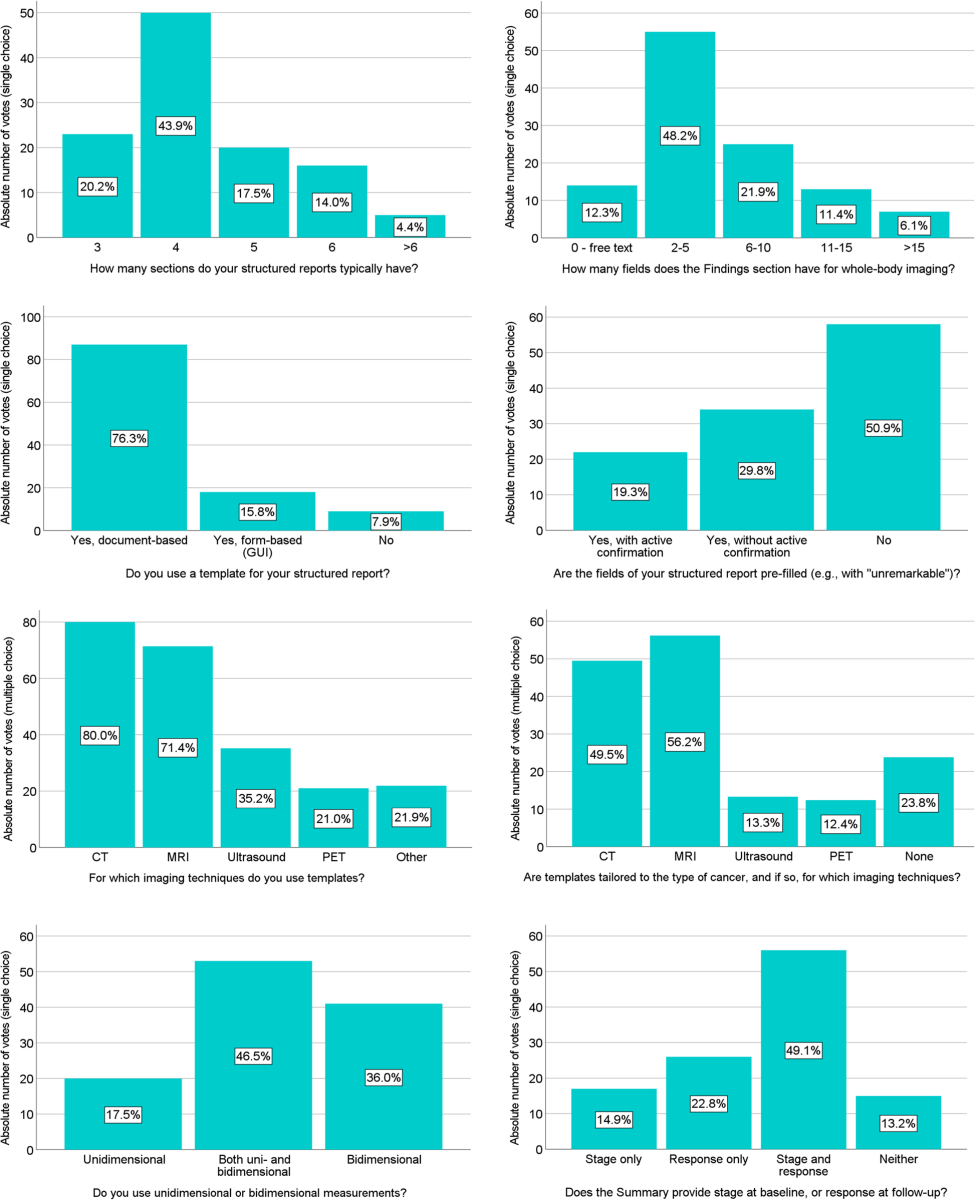
Responses of structured report users regarding the design of structured reports and templates

This survey section was exclusively completed by current SR users (*n* = 114). Reports typically had four sections, such as Indication, Technique, Findings, and Summary (43.9%, *n* = 50), with the Findings section of whole-body cross-sectional imaging reports most frequently comprising 2–5 fields/anatomic regions (48.2%, *n* = 55) or 6–10 fields/regions (21.9%, *n* = 25) (Fig. 1, ESM). The vast majority of SR users worked with templates (92.1%, *n* = 105), which were typically document-based (76.3%, *n* = 87) and without pre-filled fields (50.9%, *n* = 58). Most template users worked with tailored cancer-specific templates (76.2%, *n* = 80), especially for MRI (56.2%, *n* = 59) and CT (49.5%, *n* = 52). Types of cancers for which tailored templates were used by cancer-specific template users (*n* = 80) are shown in Fig. 1. A small majority of radiologists using SR did not utilize standardized language (e.g., for number of lesions or likelihood of malignancy) (51.8%, *n* = 59). SR users typically applied both uni- and bidimensional measurements (46.5%, *n* = 53), and provided both tumor stage and treatment response categories in their reports (49.1%, *n* = 56) (Fig. 1).

### Impact of structured reporting

**Fig. 2:**
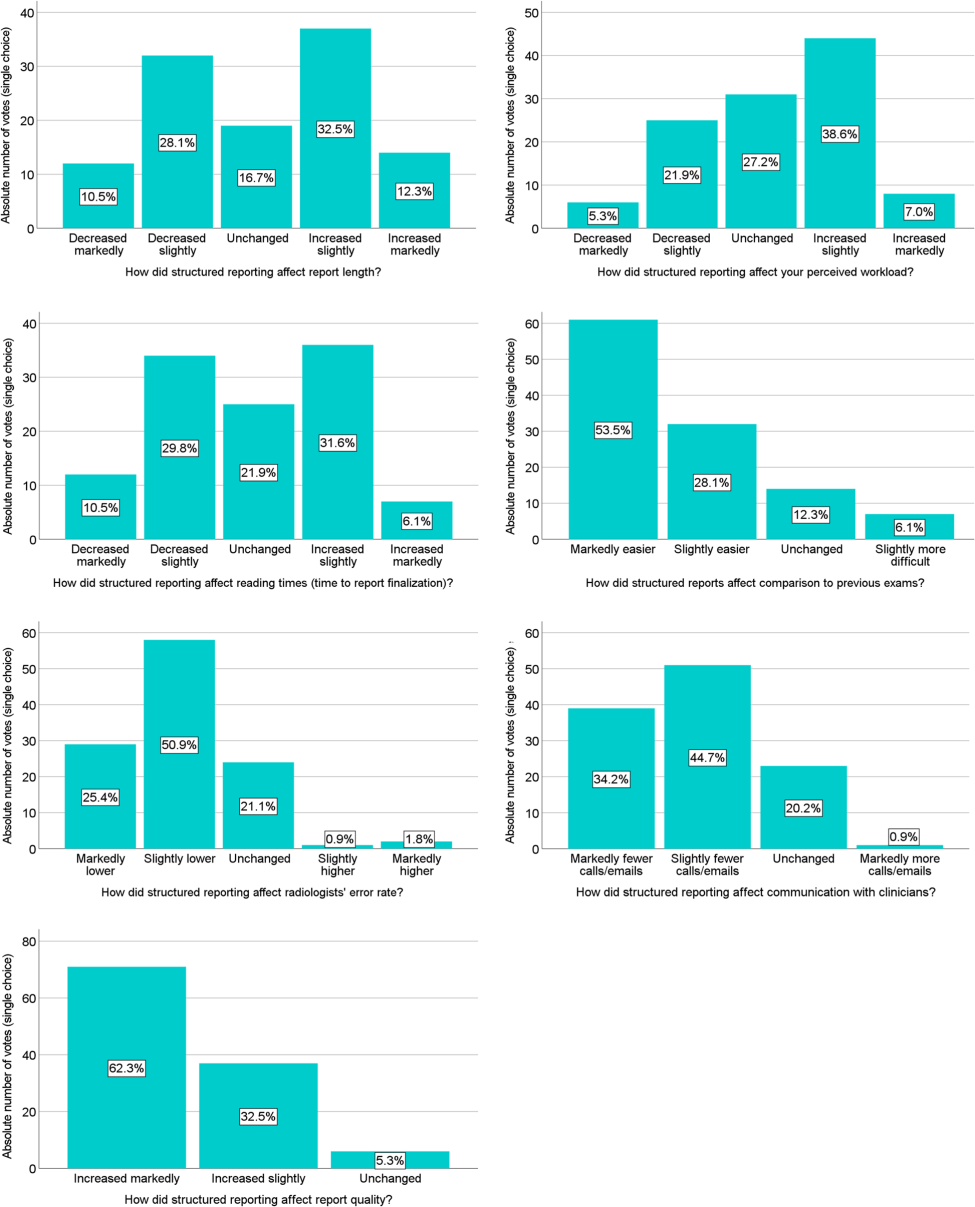
Responses of structured report users regarding the impact of structured reporting

Among current SR users (*n* = 114), the majority regarded SR favorably, stating that it markedly increased report quality (62.3%, *n* = 71), and made the comparison to previous exams markedly easier (53.5%, *n* = 61) (Fig. 2). Most participants stated that SR led to a slightly lower error rate (50.9%, *n* = 58), and fewer calls or emails by clinicians (78.9%, *n* = 90). Nevertheless, many radiologists felt that SR slightly increased radiologists’ perceived workload (38.6%, *n* = 44). Radiologists’ opinions were split regarding the impact of SR on report length and reading times (Fig. 2).

Workplace—i.e., academic vs. non-academic—did not lead to differences in the perceived impact of SR on report quality (*p* = 0.43), report length (*p* = 0.22), workload (*p* = 0.07), reading times (*p* = 0.17), comparison to prior (*p* = 0.64), error rate (*p* = 0.92), or communication with clinicians (*p* = 0.41).

Radiologists’ experience (years in practice as an ordinal variable) showed a low but significant correlation with report quality (*r* = 0.19, *p* = 0.043), perceived error rate (*r* = − 0.22, *p* = 0.016), and communication with clinicians (frequency of calls/emails) (*r* = − 0.27, *p* = 0.003). For communication with clinicians, the perceived impact of SR differed significantly between experience levels (*p* < 0.001), but not for comparison to prior exams (*p* = 0.056), or for report quality (*p* = 0.080), report length (*p* = 0.83), workload (*p* = 0.52), reading times (*p* = 0.23), or error rate (*p* = 0.15) (see Fig. 2).

Template users and non-template users differed regarding the perceived impact of SR on report quality (*p* = 0.036), with a higher percentage of template users reporting markedly improved report quality due to SR (Fig. 2). Template use did not affect perceptions of report length (*p* = 0.40), workload (*p* = 0.71), reading times (*p* = 0.85), error rate (*p* = 0.13), communication with clinicians (*p* = 0.58), or comparison to prior reports (although only minimally above the significance level, at *p* = 0.057).

Standardized language users’ opinions did not significantly differ from other radiologists with regard to report quality (*p* = 0.54), report length (*p* = 0.75), perceived workload (*p* = 0.11), reading times (*p* = 0.80), comparison to prior (*p* = 0.30), error rate (*p*= 0.36), or communication with clinicians (*p* = 0.56).

### Tailored oncologic structured report

**Fig. 3:**
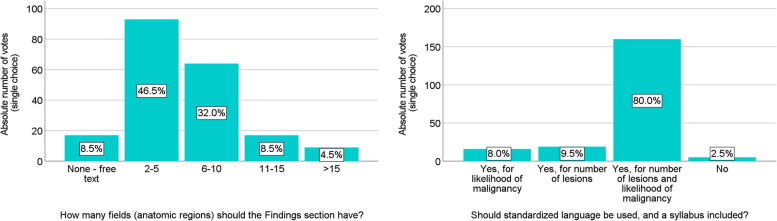
Responses of all survey participants regarding suggestions for an ESOI structured report template for CT chest/abdomen/pelvis

Study participants had the following preferences for a tailored oncologic reporting template (Fig. 3):

1. Fields/anatomic regions in the Findings section: 2–5 (46.5%, *n* = 93) or 6–10 (32.0%, *n* =64)
2. Standardized language: yes, with a syllabus for the number of lesions and likelihood of malignancy (80.0%, *n* =160)
3. Tumor stage for baseline scans: yes (87.5%, *n* = 175)
4. Treatment response category for follow-up scans: yes (89.5%, *n* = 179)
5. Inclusion of baseline data for comparison/response assessment: yes (93.5%, *n* = 187)

Based on these responses, and because there was no > 50% majority regarding the number of anatomic regions in the Findings section, two versions of an oncologic reporting template endorsed by ESOI—with four and ten anatomic regions, respectively—were created (Electronic Supplementary Material). Note that the likelihood of malignancy is based on a recently published lexicon for oncologic imaging, which uses five categories for diagnostic certainty (“unlikely,” “less likely,” “possibly,” “probably,” and “consistent with”) and three categories for numbers of lesions (“few,” “multiple,” and “numerous”) [26].

## Discussion

Imaging plays an essential role in the complex task of high-quality cancer care, and as such, can be expected to profit from the growing implementation of SR, for the benefit of radiologists, clinicians, and patients alike. To our knowledge, this is the first study to assess radiologists’ current use of and perceptions of SR in an exclusively oncologic imaging setting.

SR was used by 57% of our survey respondents, which is a higher proportion as compared to prior studies [16, 27], and might reflect the slowly but steadily growing acceptance and application of SR worldwide—or, alternatively, a greater interest in SR by oncologic imaging specialists. In the latter case, this may be due to the fact that cancer frequently presents as a multi-organ disease, and SR facilitates an overview of its spread across the body. Notably, European radiologists were significantly less likely to use SR in their daily practice compared to non-Europeans, a finding that could not be explained by the slightly higher percentage of academic radiologists in the European subgroup, as the use of SR did not differ significantly between work settings. Therefore, this finding clearly highlights the need for further SR promotion by specialized imaging societies, such as ESOI, or on a higher level, the ESR, to avoid a future disadvantage in terms of quality and competitiveness of European radiologists and healthcare institutions.

Our survey participants generally approved of SR use, stating that it markedly increased report quality, and made comparison of serial exams markedly easier, leading to a slightly lower error rate, and better communication with clinicians. In a large Italian cohort, Faggioni et al showed that radiologists overall were in favor of SR implementation, mentioning advantages such as reproducibility, and interaction with clinicians [16]. Their data from radiologists of a single country and society are concordant with findings in our Europe-based, but international oncologic imaging society. Despite our otherwise clearly positive results, SR slightly increased radiologists’ perceived workload—a finding that may be critical in view of the increasing numbers of imaging exams [20], workforce shortage [28], and risk of burnout [21] that radiologists face. In prior studies, SR was mostly criticized for rigidity, inefficiency, simplification, and detrimental effects on resident education [16, 27, 29], while an impact on workload has not been demonstrated so far, and might therefore be related to oncologic imaging as a subspecialty. The latter could also potentially explain the discrepancy between increased workload and unchanged reading times, as well as easier comparison and better communication in our study, which might stem from the limited suitability of SR to complex cases (which occur frequently in oncology), or loss of report coherence, as previously suggested by Powell et al [27]. Another possible explanation could be insufficient experience with SR, e.g., a recent switch from traditional free-text reporting to SR. Importantly, the use of SR was only considered to *slightly* increase the workload, which, in the authors’ experience, may amount to +5 minutes per report, depending on the extent of the disease. Still, strategies to tackle increased radiologist workload should be considered. If the perceived workload increase is indeed transient, the required time interval to get used to SR could probably be shortened with dedicated SR training. On the reporting level, one strategy may be to limit the number of measured lesions per anatomical region/organ, because measurements, especially when bidimensional, are clearly time-consuming. In this context, research investments to drive technical innovations, such as automated or semi-automated AI-based lesion segmentation and tumor burden quantification tools, may play an important role in the future, and help address workforce shortage and radiologist wellbeing [30].

Template use was significantly associated with a perceived increase in report quality in our study, and a near-significant improvement in comparison with prior exams. This finding agrees with previous studies, for example, Brown et al and Andersen et al, where radiologists and clinicians rated template style reports as superior to free text about key findings in a single cancer and imaging modality [15, 31]. Our results suggest that document-based templates are currently favored over graphical user interface-based templates, possibly because this solution is technically less demanding and easier to integrate with current speech recognition systems, which are also document-based to facilitate corrections by the radiologist. Our data revealed that there is currently no consensus on whether template fields should be pre-filled (e.g., with “unremarkable”) or not. While pre-filled fields guarantee report completeness because the risk of missing entries is eliminated, it may increase the risk of “overlooking” individual organs/tissues due to an existing entry, especially when no active confirmation of the pre-filled entry is required.

Cancer-specific templates appear to be already quite popular, especially for MRI, where 56% of template users already apply them routinely (Fig. 1). This is not surprising, given the demand by surgeons and oncologists to include specific details that are relevant for their respective decision making and treatment planning in imaging reports. An example of the latter would be rectal cancer, where the exact anatomic location and extent of the tumor within the rectum directly affect the type of surgery. Such additions or modifications of structured reports, which can also be used to facilitate reporting of critical findings, are increasingly implemented in radiology under the term “contextual reporting,” with the aim to ensure report completeness from a clinical perspective [32].

Standardized language, utilized by 48% of SR users in our study, was not associated with any of the assessed variables of perceived clinical impact, neither positively nor negatively. Still, our survey participants have a very clear preference for standardized language in radiology reports. The latter is also essential for data mining and AI-based NLP [13], and therefore, we recommend its use. NLP can for instance be used to assist radiologists in generating high-quality reports by automatically identifying and communicating key and critical findings, lesion measurements (including changes in tumor size over time), and relevant clinical information [22]. For instance, Spandorfer et al used deep learning methods to convert unstructured CT chest reports into SR [33], while Yen et al. utilized a dual-AI to detect lung nodules and determine, using NLP, whether these lung nodules were included in the report [34]. Possible future applications include the auto-population of templates with recommendations and follow-up suggestions, for instance in terms of recommended imaging tests for further evaluation, or follow-up intervals according to published guidelines; or the reduction of errors by double-checking measurements and associated SI units (e.g., cm vs. mm), or by laterality matching between Findings and Impression sections; or workload reduction, for instance by automatic insertion of technical parameters, such as type of injected contrast agent or radiotracer, and their respective doses. Such applications can be expected to improve overall reporting efficiency.

In our survey, more senior/experienced radiologists stated that SR positively affects communication with clinicians, and results in fewer calls or emails, a finding that can probably be explained by the longer observation period that makes abrupt changes in clinicians’ behavior more obvious, and a long-standing work relationship and closer communication with clinicians. There was also a trend towards more experienced radiologists thinking that SR improves comparison to previous scans, as well as report quality, possibly due to advances in knowledge and a better understanding of clinicians’ needs, reflecting professional growth.

Our study is limited by its moderate number of participants who were recruited from a single imaging subspecialty society (ESOI) based in Europe. Due to this inherent selection bias, 72.5% of respondents were European, which clearly limits the international generalizability of results. The imbalance in geographical distribution within the society—78% of active ESOI members in 2022 were European—together with the time point chosen for our survey, may also largely explain the rather low response rate of 25% overall (23% for European respondents, and 31% for international respondents), as July and August are the traditional summer vacation months in many European countries. While we were aware of the latter, we had assumed that this would be an advantage, because radiologists may be more likely to respond due to the decrease in routine clinical work pressure, which was probably incorrect. In addition, ESOI is an academic society, and therefore, there might be an inherent selection bias towards academic institutions; however, 42.5% of respondents did not work in an academic setting, so any such effect was probably limited. While we assessed radiologists’ experience level as years of radiology practice, we did not assess years of experience with SR, which, in hindsight, might have been an interesting aspect. No oncologists were invited to participate in our study because we aimed to specifically address radiologists’ current practice and implications of SR on their workflow; still, a clinician’s view on SR may have offered additional insight, and may therefore be subject to further research.

In conclusion, radiologists generally appreciated SR for cancer imaging, noting, in particular, its positive effects on report quality, error rate, comparison of serial exams, and communication with clinicians, at the expense of a slightly increased workload. The latter finding needs to be addressed further in a comprehensive effort of the imaging community, especially in light of steadily increasing scan volumes. The lower utilization rate of SR by European radiologists compared to their non-European colleagues clearly also needs to be tackled by the ESR, in collaboration with the European national radiology societies under its umbrella. As an ESR subsociety, ESOI has set its goal of offering dedicated workshops as part of its annual meetings, to further promote the use of SR, given its overall very positive impact on reporting.

### Supplementary Information

Below is the link to the electronic supplementary material.

Supplementary file1 (PDF 155 KB)

Supplementary file2 (PDF 56.1 KB)

## Abbreviations

AI: Artificial intelligence
ESOI: European Society of Oncologic Imaging
ESR: European Society of Radiology
RSNA: Radiological Society of North America
SR: Structured reporting

## Declarations

### Guarantor

The scientific guarantor of this publication is Marius E. Mayerhoefer.

### Statistics and biometry

No complex statistical methods were necessary for this paper.

### Methodology

• multicenter study
