# Supplementary material for: Perceptions of radiologists on structured reporting for cancer imaging—a survey by the European Society of Oncologic Imaging (ESOI)
Source: Eur Radiol. 2024 Jan 11;34(8):5120–30. doi: 10.1007/s00330-023-10397-6 (PMC11254975; doi:10.1007/s00330-023-10397-6)
Supplement: Supplementary file 1 — Supplementary file1 (PDF 155 KB) [file 330_2023_10397_MOESM1_ESM.pdf]

# Perceptions of radiologists on structured reporting for cancer imaging – a survey by the European Society of Oncologic Imaging (ESOI)

## Electronic Supplementary Material

*Eur Radiol (2023) Leithner D, Sala E, Neri E et al.*

**CT CHEST, ABDOMEN, PELVIS**

**DATE:**

**CLINICAL INDICATION:**

**TECHNIQUE:**

**COMPARISON TO:**

**FINDINGS:**

LUNGS AND PLEURA:

MEDIASTINUM / THORACIC NODES:

HEPATOBILIARY:

PANCREAS:

SPLEEN:

KIDNEYS AND ADRENAL GLANDS:

ABDOMINOPELVIC NODES:

GASTROINTESTINAL TRACT / PERITONEUM:

PELVIC ORGANS:

BONES AND SOFT TISSUES:

**CONCLUSION:**

**TUMOR STAGE (BASELINE):**

**TREATMENT RESPONSE:**

☐CR ☐PR ☐SD ☐PD ☐N/A ☐Other:

<u>Diagnostic certainty:</u>

| | |
|---|---|
| Unlikely: | <10% |
| Less likely: | ~25% |
| Possibly: | ~50% |
| Probably / suspicious for: | ~75% |
| Consistent with: | >90% |

<u>Quantitative descriptors:</u>

| | |
|---|---|
| Few: | 2-5 |
| Multiple : | 6-20 |
| Numerous: | >20 |

**CT CHEST, ABDOMEN, PELVIS**

European Society of Oncologic Imaging

**DATE:**

**CLINICAL INDICATION:**

**TECHNIQUE:**

**COMPARISON TO:**

**FINDINGS:**

CHEST:

ABDOMEN:

PELVIS:

BONES / SOFT TISSUES:

**CONCLUSION:**

**TUMOR STAGE (BASELINE):**

**TREATMENT RESPONSE:**

☐CR ☐PR ☐SD ☐PD ☐N/A ☐Other:

<u>Diagnostic certainty:</u>

| | |
|---|---|
| Unlikely: | <10% |
| Less likely: | ~25% |
| Possibly: | ~50% |
| Probably / suspicious for: | ~75% |
| Consistent with: | >90% |

<u>Quantitative descriptors:</u>

| | |
|---|---|
| Few: | 2-5 |
| Multiple : | 6-20 |
| Numerous: | >20 |
