# Supplementary figures and images for: Perceptions of radiologists on structured reporting for cancer imaging—a survey by the European Society of Oncologic Imaging (ESOI)
Source: Eur Radiol. 2024 Jan 11;34(8):5120–30. doi: 10.1007/s00330-023-10397-6 (PMC11254975; doi:10.1007/s00330-023-10397-6)

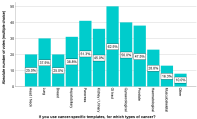

Supplement: Supplementary file 2 — Supplementary file2 (PDF 56.1 KB) [file 330_2023_10397_MOESM2_ESM.pdf]
